# Supplementary material for: Mapping the learning curves of deep learning networks
Source: PLoS Comput Biol. 2025 Feb 10;21(2):e1012286. doi: 10.1371/journal.pcbi.1012286 (PMC11841907; doi:10.1371/journal.pcbi.1012286)
Supplement: S1 Text — Sentence and gesture RNN-LSTM model performance. (PDF) Table A. Average Performance metrics for sentence classification across all simulation runs (epochs = 50). (PDF) Table B. Average Performance metrics for gesture classification across all simulation runs (epochs = 50). (PDF) Table C. Average performance metrics for 10 examples of gesture classification across all simulation runs (epochs = 100). (PDF) [file pcbi.1012286.s001.pdf]

S1 Text. Sentence and gesture RNN-LSTM model performance. The model performance for the average of all pairwise simulations of sentence and gesture classification in the main text (epochs = 50), including metrics such as validation accuracy, validation loss, test accuracy, test loss, recall, precision, and F1. These results confirm that almost all pairwise classifications (except for body & head) have been sufficiently trained, indicating that the learning curves are capturing the learned experiences, with sentence classification achieving slightly better performance than gesture classification. Regarding the body & head classification, this corresponds to the unique learning trajectory of the underlying pair, which struggles to properly learn the signals. This has been discussed in our manuscript. Additionally, we extended gesture classification to 100 epochs for 10 pairwise simulations to showcase that, even for more difficult learning tasks, the performance and underlying learning remain largely consistent with the patterns we illustrated for epochs = 50 in our manuscript. The visualization of this analysis can be found in S4 Text.

Table A. Average Performance metrics for sentence classification across all simulation runs (epochs=50).

Pairwise Classes	Val Acc	Val Loss	Test Acc	Test Loss	Recall	Precision	F1
Anger & Fear	86.3535	0.0015	86.0045	0.0016	0.8980	0.8713	0.8771
Anger & Joy	95.1965	0.0005	95.2182	0.0010	0.8517	0.8782	0.9049
Anger & Love	87.9834	0.0020	84.7724	0.0030	0.7581	0.7351	0.7824
Anger & Sadness	89.0694	0.0012	84.3187	0.0022	0.8350	0.7790	0.7845
Anger & Surprise	99.5154	0.0001	97.9689	0.0006	0.8964	0.9196	0.9067
Joy & Fear	90.4452	0.0013	84.8238	0.0036	0.9066	0.9069	0.9058
Joy & Sadness	70.1222	0.0024	68.3128	0.0024	0.6550	0.7867	0.7261
Joy & Surprise	67.8336	0.0027	66.4760	0.0035	0.5805	0.5899	0.5082
Love & Fear	92.3434	0.0007	90.2093	0.0011	0.9031	0.9510	0.9054
Love & Joy	83.2770	0.0020	79.4084	0.0026	0.8321	0.7669	0.7725
Love & Sadness	70.6044	0.0031	69.3720	0.0037	0.7920	0.6818	0.7181
Love & Surprise	90.5970	0.0011	83.3547	0.0026	0.8522	0.7187	0.7549
Sadness & Fear	97.9751	0.0004	96.5514	0.0007	0.9734	0.9797	0.9762
Surprise & Fear	97.0638	0.0004	95.4549	0.0010	0.9696	0.9797	0.9745
Surprise & Sadness	78.5151	0.0024	74.3526	0.0030	0.7850	0.8665	0.7842
Null	58.4898	0.003	55.1542	0.0039	0.4648	0.4008	0.3744

Table B. Average Performance metrics for gesture classification across all simulation runs (epochs=50).

Pairwise Classes	Val Acc	Val Loss	Test Acc	Test Loss	Recall	Precision	F1
Body & Body-Head	84.375	0.0030	80.3811	0.004	0.7661	0.6104	0.6773
Body & Hand	85.5880	0.0028	83.6798	0.0032	0.8221	0.7579	0.7858
Body & Head	50.8064	0.0056	53.8326	0.0059	0.4023	0.3449	0.3022
Body & Head-Hand	95.3139	0.0012	94.4681	0.0016	0.9443	0.9014	0.9220
Body & No Gestures	79.1063	0.0040	78.1145	0.0040	0.7858	0.8253	0.8029
Body-Head & Hand	87.2161	0.0030	85.1621	0.0035	0.8611	0.8968	0.8775
Body-Head & Head	83.195	0.0035	81.895	0.0035	0.8229	0.8635	0.8415
Body-Head & Head-Hand	84.4321	0.0031	83.8148	0.0033	0.8853	0.8482	0.8651
Body-Head & No Gestures	90.0473	0.0023	84.3183	0.0041	0.8834	0.9151	0.8985
Head & Hand	82.3948	0.0037	81.9935	0.0035	0.8485	0.8197	0.8325
Head & Head-Hand	94.2811	0.0016	94.0976	0.0015	0.9426	0.9419	0.9420
Head & No Gestures	83.2581	0.0034	79.1281	0.0042	0.8013	0.8983	0.8462
Head-Hand & Hand	94.8785	0.0014	94.4766	0.0014	0.9421	0.9523	0.9469
Head-Hand & No Gestures	94.7905	0.0013	93.2634	0.0018	0.9354	0.9701	0.9523
No Gestures & Hand	88.1993	0.0028	84.4616	0.0035	0.7932	0.7291	0.7565
Null	52.7253	0.0058	48.9855	0.0059	0.5607	0.4222	0.4304

Table C. Average performance metrics for 10 examples of gesture classification across all simulation runs (epochs = 100).

Pairwise Classes	Val Acc	Val Loss	Test Acc	Test Loss	Recall	Precision	F1
Body & Body-Head	88.0441	0.0032	81.073	0.0062	0.7118	0.6237	0.6639
Body & Hand	87.2742	0.003	84.470	0.004	0.8113	0.7806	0.7942
Body & Head	52.3522	0.0056	46.5504	0.006	0.6416	0.3492	0.4345
Body & Head-Hand	95.4417	0.0013	94.6048	0.0016	0.9457	0.9049	0.9246
Body-Head & Hand	89.6739	0.0032	86.2843	0.0044	0.8926	0.8827	0.8873
Body-Head & Head	84.5093	0.0040	82.1804	0.0042	0.8435	0.8499	0.8463
Body-Head & Head-Hand	87.0432	0.0034	84.1931	0.0044	0.8865	0.8484	0.8664
Head & Hand	82.7292	0.0041	82.4811	0.0041	0.8476	0.8292	0.8363
Head & Head-Hand	94.2823	0.0017	94.3814	0.0015	0.9488	0.9438	0.9461
Head-Hand & Hand	94.6655	0.0016	94.5425	0.0016	0.9487	0.9486	0.9486
