# Supplementary material for: Mapping the learning curves of deep learning networks
Source: PLoS Comput Biol. 2025 Feb 10;21(2):e1012286. doi: 10.1371/journal.pcbi.1012286 (PMC11841907; doi:10.1371/journal.pcbi.1012286)

S2 Text. Illustrations of multiclassification results for sentence classification (epochs=20). This section demonstrate that our method can be easily adapted to multiclassification tasks (e.g., learning to distinguish between six emotions in sentence classification) as well as to binary comparisons within a multiclass classification task (e.g., anger vs. sadness, fear vs. joy). These additional simulations for sentence classification tasks illustrate that, with the presence of other classes, the learning experience of binary classification differs from the purely pairwise classification we presented in the main text. Specifically, the learning tends to converge to more coherent performance and patterns, with less stochasticity observed.

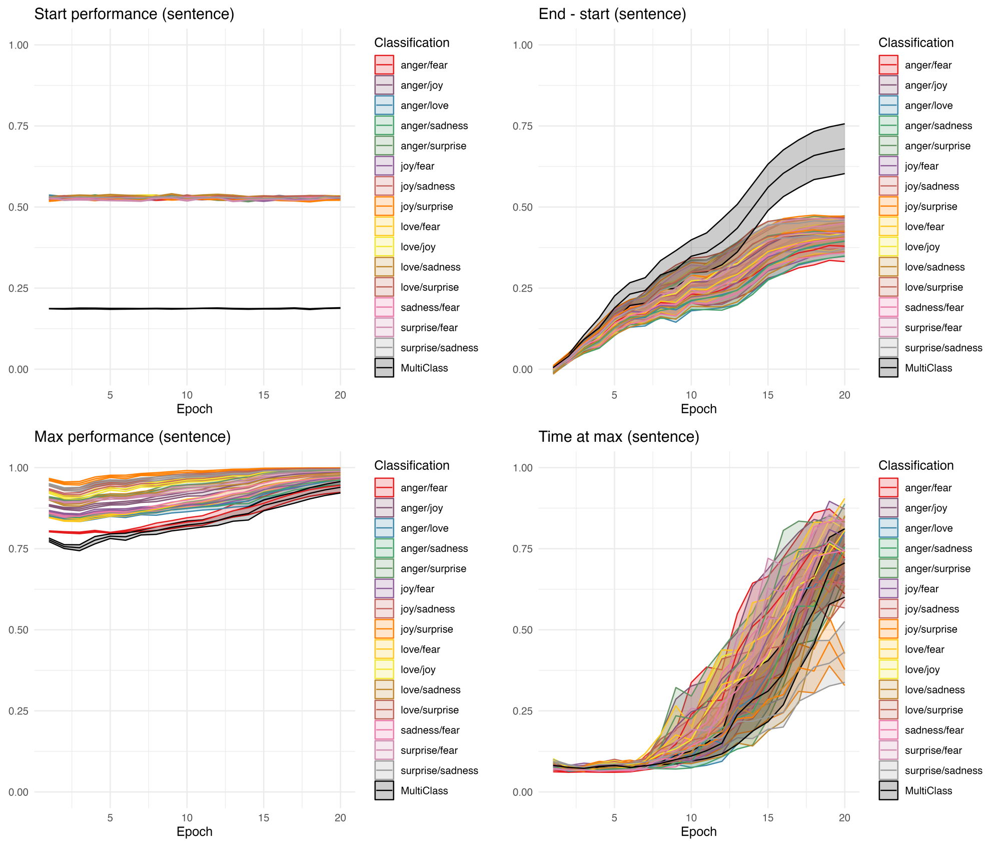

Supplement: S2 Text — Illustrations of multiclassification results for sentence classification (epochs = 20). (PDF) [file pcbi.1012286.s002.pdf]
