# Supplementary material for: Mapping the learning curves of deep learning networks
Source: PLoS Comput Biol. 2025 Feb 10;21(2):e1012286. doi: 10.1371/journal.pcbi.1012286 (PMC11841907; doi:10.1371/journal.pcbi.1012286)
Supplement: S3 Text — Illustrations of sentence and gesture simulations across 20 epochs. (PDF) [file pcbi.1012286.s003.pdf]

S3 Text. Illustrations of sentence and gesture simulations across 20 epochs. These simulations aim to demonstrate that, for our learning curve method, which focuses more on the early developmental experience (epochs < 50) due to its richer qualitative insights, analyzing simulations at epochs $= 20$ reveals very similar patterns to the epochs $= 50$ simulation we illustrated in the manuscript.

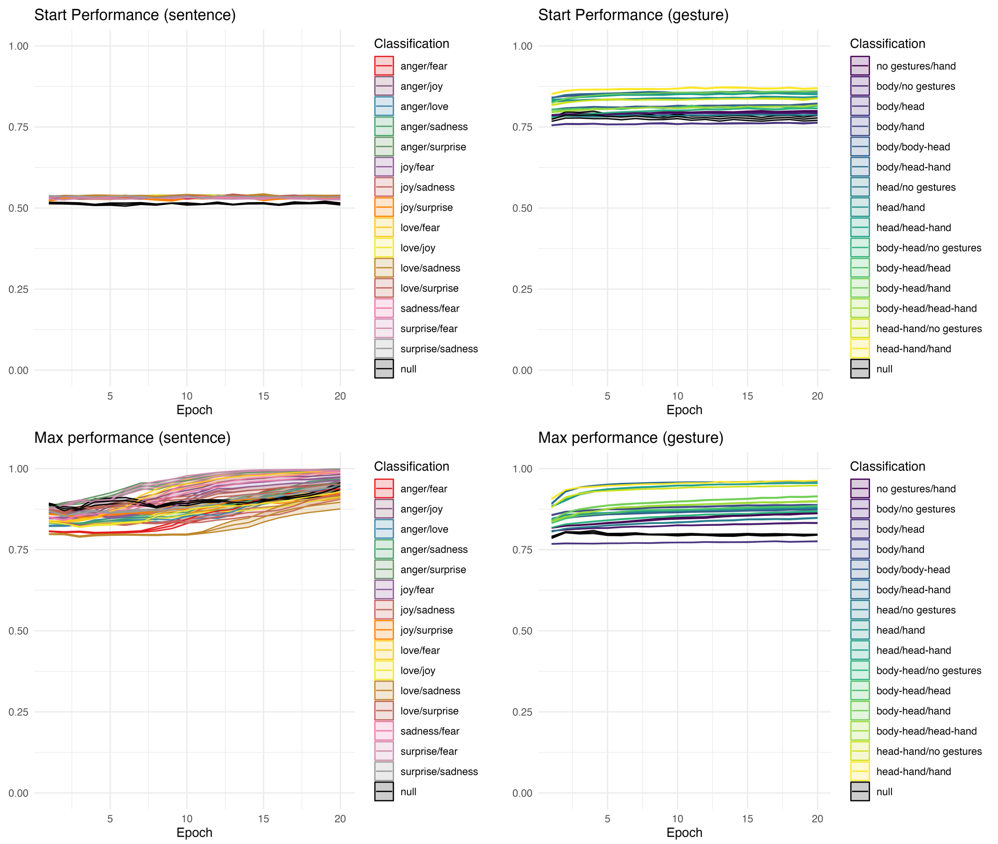

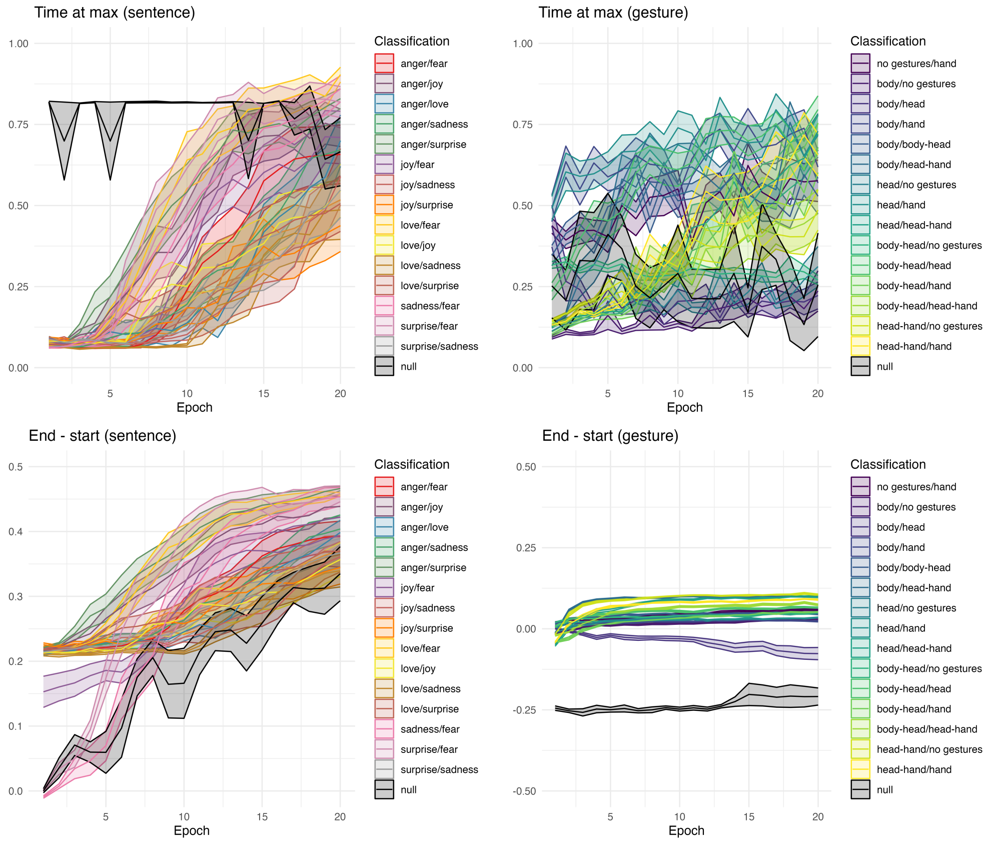
Time at max (sentence)
Classification
anger/fear
anger/joy
anger/love
anger/sadness
anger/surprise
joy/fear
joy/sadness
joy/surprise
love/fear
love/joy
love/sadness
love/surprise
sadness/fear
surprise/fear
surprise/sadness
null
Epoch
Time at max (gesture)
Classification
no gestures/hand
body/no gestures
body/head
body/hand
body/body-head
body/head-hand
head/no gestures
head/hand
head/head-hand
body-head/no gestures
body-head/head
body-head/hand
body-head/head-hand
head-hand/no gestures
head-hand/hand
null
Epoch
End - start (sentence)
Epoch
End - start (gesture)
Epoch
