# Supplementary material for: Mapping the learning curves of deep learning networks
Source: PLoS Comput Biol. 2025 Feb 10;21(2):e1012286. doi: 10.1371/journal.pcbi.1012286 (PMC11841907; doi:10.1371/journal.pcbi.1012286)

S4 Text. Illustrations of gesture simulations across 100 epochs. Given that gesture learning could be more difficult than sentence classification, we further ran 10 pairwise simulations for the gesture learning task to see if the learning curves resulted in qualitatively distinct patterns. These simulations confirm that extending to 100 epochs produced similar patterns, as most of the learning variation occurs in the relatively early phase when epochs are smaller than 50.

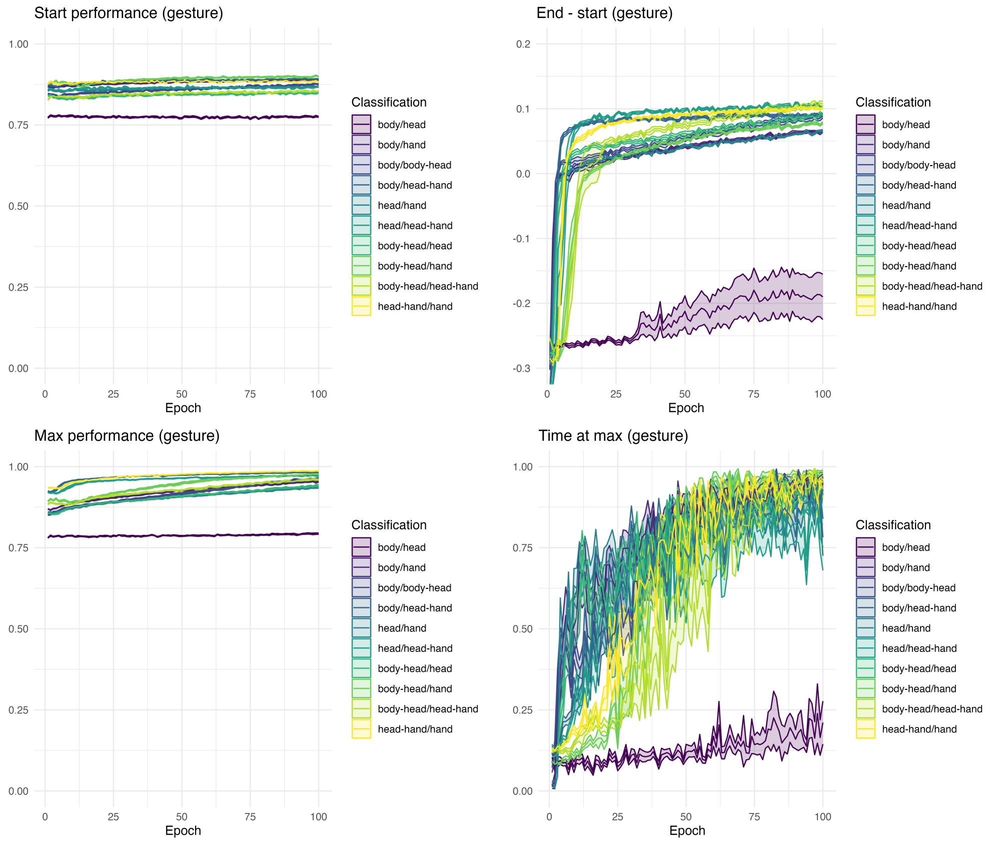

Supplement: S4 Text — Illustrations of gesture simulations across 100 epochs. (PDF) [file pcbi.1012286.s004.pdf]
