# Supplementary material for: Mapping the learning curves of deep learning networks
Source: PLoS Comput Biol. 2025 Feb 10;21(2):e1012286. doi: 10.1371/journal.pcbi.1012286 (PMC11841907; doi:10.1371/journal.pcbi.1012286)
Supplement: Attachment — Submitted filename: Review.pdf [file pcbi.1012286.s005.pdf]

Review comments for PCOMPBIOL-D-24-01100
"Mapping the Learning Curves of Deep Learning Networks"

This paper investigated the "learning curves" of deep learning networks, specifically recurrent neural networks. For the training process, each input is a multi-dimensional time series. The learning curves refer to the classification accuracy at different timesteps of the series within one epoch, or at the same timestep of the series across different training epochs. By analyzing the learning curves on two tasks, gesture classification and emotion tweet classification, the authors summarized some interesting insights.

Overall, this paper is great, with detailed descriptions and easy-to-follow English. I only have a few comments before its publication.

- The gesture classification and emotion tweet classification both consist of 6 classes (after grouped for gesture dataset). However, the models are designed for binary classification between each pair of classes. Although insights can be drawn from the binary classification results, is it easy to apply these insights to multi-class classifications? If so, it would be better to adjust the models to classify all inputs directly into their respective classes.
- The abbreviation "DNN" in the abstract should be expanded to its full form the first time it appears.
- In the conceptual illustration in Fig. 1, the curves for all epochs start at the same points. Although the results in Fig. 4 may support this, it is initially confusing. I suggest either plotting with slight differences at the start points or providing an explanation that at the start of the timestep, the information from the time series is very low.
- In lines 185 and 214, the time series are originally of different lengths and were padded to a consistent length for both NLP and Gesture data. Could the type of padding methods be added?
- In line 284, it is great to provide the hyperparameter learning rate. Were other hyperparameters, like the betas in Adam, kept at their default values?
- In line 288, why does a batch size of 1 increase the total amount of input data? In my understanding, even for batch sizes larger than 1, all the input data would be fed into the model. Could you clarify this?
- In line 339, the definition of t_{max}. should be more accurate. It is defined as the time bin, which I understand should be between 1,...,66 for NLP and 1,...,150 for Gesture. However, in Fig. 3, it seems to be the percentage over the whole timesteps as it is in $[0, 1]$.
- In line 370, "Across epochs," should be "Within a epoch,".
- I checked the code in the GitHub repository and found an error near the end of the notebook Gesture Classification_pairwise_sim_allEpoch.ipynb. Additionally, I noticed many warnings about "Maximum iterations reached" and the optimization not converging yet. Does this affect the results?
- In line 523, there is a missing closing parenthesis for a corresponding opening parenthesis.
