# Supplementary material for: Mapping the learning curves of deep learning networks
Source: PLoS Comput Biol. 2025 Feb 10;21(2):e1012286. doi: 10.1371/journal.pcbi.1012286 (PMC11841907; doi:10.1371/journal.pcbi.1012286)
Supplement: Attachment — Submitted filename: Opinion_PLOS Computational Biology.pdf [file pcbi.1012286.s006.pdf]

PLOS Computational Biology

Sobre o artigo: PCOMPBIOL-D-24-01100Mapping the Learning Curves of Deep Learning Networks

The authors of this research present a set of techniques that can quantify and visualize two temporal vector inputs (two temporal dimensions thus characterized) and apply models known as Deep Neural Network, Natural Language Processing, Normalization, Long-Term Memory, among others. The study proposes an interpretation model that extracts learning curves.

Regarding the proposal.

Positive points: the manuscript follows a didactic sequence and the entire process of the techniques is described in an accessible way. The study also tests on a good validation sample.

However, some points were not clear in the writing. They are:

1- Abstract:

Describes what “natural language processing” means and stands for (NLP). Why didn’t you do the same with Deep Neural Networks (DNN)?

2- The current study:

To conduct such a study and unpack “DNN learning curves, ...

It was not clear what some understandings are in this item:

i) Ability to process time series, ...

ii) Capture temporal dependencies through their recurrent connections, ...

iii) Use as a basis changes in “predictability” or “information”, ...

This information was not clear.

Subjectivity in the format of how this process occurs.

My suggestion is a more detailed description of the generation of the series and how this information is captured.

3- Feedforward network:

A normalization was applied to the input. It was conducted in every time interval. Normalization was applied to the input to convert information, ...

There are many details in the text about normalization, however, the technique is vague. The text does not describe that normalization is used to transform input data so that it is within a domain (specific range) with a focus on improving the network's performance. We have normalization for training, to prevent or fade gradients to improve performance and we also have types of normalization.

The question goes in this direction. What is the objective (to describe)? What is the type (to describe)? What is the domain of normalization? 0 and 1?

I understand that this can be done. It would help to better understand this topic.

4- Quantifying performance across epochs

The description of this topic was unclear on some points:

i) We tested several regression techniques. Which ones? Technically, we have several. It was a simple regression, logistic, polynomial, Ridge, Poisson, Support Vectors, Bayesian, ... I believe you can tell us which regression was used.

ii) The regression coefficients. Which ones?

iii) Simple summary statistics. Do you mean average? What is this simple summary? I understand that you can add details.

iv) What would explain the transition of environment (Python to R)? What is Python limited in?

5- Results

Again, I repeat here. We tested a linear model. Which one?

6- Discussion

Check for non-linearity (it's in the text).

I understand that you can add details on how you checked for non-linearity.

7- Domain distinctions

To characterize the smoothness of a curve from the point of view of autocorrelation, it is very common to use a model that takes the scale self-affinity, a property of looking at information from the point of view of time scales, for example the Detrended Fluctuation Analysis method, Peng 1994.

For the information described “Smoother self-correlated signals”, I actually didn’t understand. Another point is also related to “self-correlated spatial information contained in the gesture performance. I believe more details can be added to this description.
