# Supplementary material for: Mapping the learning curves of deep learning networks
Source: PLoS Comput Biol. 2025 Feb 10;21(2):e1012286. doi: 10.1371/journal.pcbi.1012286 (PMC11841907; doi:10.1371/journal.pcbi.1012286)
Supplement: Attachment — Submitted filename: response to reviewers.pdf [file pcbi.1012286.s007.pdf]

Dear Editors and Reviewers,

We would like to express our sincere gratitude to you for such insightful and constructive comments on our manuscript. We appreciate the time and effort you've devoted to evaluating our work and providing valuable feedback that has greatly improved the quality of our paper. We believe that the end product is in a better shape compared to our previous version.

In this response, we attached each reviewer's comment, addressed each comment in detail and explained how we have revised our manuscript accordingly. We hope that the revisions we have made will satisfy the reviewers and enhance the contribution of our work to the field.

In this round of revision, we have provided additional analysis for both the main text and supporting information to strengthen our arguments. These include extending the simulations to 50 epochs and demonstrating that the learning curves reveal similar patterns to our previous 20 epochs. We also incorporated a more meaningful superimposed (reshuffled) null hypothesis to show that the learning curves from ordered classification tasks qualitatively differ from those of the reshuffled null. This hypothesis testing further strengthens the applicability and generalizability of our method in revealing the "(un)learning experience" of reshuffled or low-quality data. We also demonstrated that our method can be easily adapted to multiclass classification analysis, with results provided in supporting information.

Additionally, we have streamlined our methods and results sections by including a meta-result table that organizes all significant tests from various regression models, and we have cited data from this table in the subsections of each metric to improve the flow of the paper.

We provide clarification on our previous "nonlinearity" insights, which more specifically refer to the observation of "non-monotonicity" in the learning curves. We have updated the theoretical implications in the discussion section on this concept accordingly.

Finally, we added clarification that, for the current scope, we aim to focus on how our proposed method can facilitate the understanding of distinguishable modalities as a first step. In the future, this method could contribute to understanding integrated multimodal processing. We have added this reflection, along with other limitations such as generalizability to other DNN model architectures, to the limitations and future research section of the discussion.

Sincerely,
The corresponding author

Reviewer 1

1. The gesture classification and emotion tweet classification both consist of 6 classes (after grouped for gesture dataset). However, the models are designed for binary classification between each pair of classes. Although insights can be drawn from the binary classification results, is it easy to apply these insights to multi-class classifications? If so, it would be better to adjust the models to classify all inputs directly into their respective classes.
   Thank you for raising this point. Our method can be easily adapted to multiclassification tasks (e.g., learning to distinguish between six emotions in sentence classification) as well as to binary comparisons within a multiclass classification task (e.g., anger vs. sadness, fear vs. joy). We have provided additional simulations for sentence classification tasks in S2 Fig. to illustrate the insights and applicability of our proposed method.

   We chose to use the original 30 binary classification simulations (15 sentence + 15 gesture) in the main text primarily because the binary learning experience from the multiclass classification task is not simply a comparison of one class against another, but rather involves the conditional effect of other classes being present. This can complicate interpretation but also presents an exciting problem to explore! Therefore, we have expanded on these insights, along with visualizations, in S2 Fig.

   Additionally, in the main text, we wanted to run enough simulations to showcase the robustness and generalizability of our method, and using binary classification directly allowed for more simulation runs as desired.

2. The abbreviation "DNN" in the abstract should be expanded to its full form the first time it appears.
   The full name has been added in the abstract.

3. In the conceptual illustration in Fig. 1, the curves for all epochs start at the same points. Although the results in Fig. 4 may support this, it is initially confusing. I suggest either plotting with slight differences at the start points or providing an explanation that at the start of the timestep, the information from the time series is very low.
   Thank you for your great suggestion. In a typical binary classification task, the starting point should be around 0.50 for all epochs (i.e., all processing curves), so we kept the same figure here and provided additional explanation. We also added clarification that for certain tasks, the starting point might differ if the data contains spatial information, such as the location of keypoints at the initial timestep.

4. In lines 185 and 214, the time series are originally of different lengths and were padded to a consistent length for both NLP and Gesture data. Could the type of padding methods be added?
   The zero-padding method has been added.

5. In line 284, it is great to provide the hyperparameter learning rate. Were other hyperparameters, like the betas in Adam, kept at their default values?
   Yes, all the learning rate hyperparameters were kept at their default values. We have added the information.

6. In line 288, why does a batch size of 1 increase the total amount of input data? In my understanding, even for batch sizes larger than 1, all the input data would be fed into the model. Could you clarify this?
   Thank you for pointing that out. You are correct; the batch size is not related to the total amount of input data we can provide. When we previously ran the simulation, because each input of the gesture recognition requires a lot of memory, we set the batch size to 1 in order to run the simulation. We have now switched to a better GPU (Nvidia RTX 4090 Ti) and optimized our code to further improve computational efficiency. As a result, we are no longer limited by this concern and have updated the batch size accordingly in the manuscript.

7. In line 339, the definition of tmax. should be more accurate. It is defined as the time bin, which I understand should be between 1,...,66 for NLP and 1,...,150 for Gesture. However, in Fig. 3, it seems to be the percentage over the whole timesteps as it is in [0, 1].
   Thank you for pointing that out. You are correct; tmax essentially represents the percentage of time across the entire set of timesteps. We have updated it accordingly.

8. In line 370, "Across epochs," should be "Within a epoch,".
   Thanks for the correction. We have updated it.

9. I checked the code in the GitHub repository and found an error near the end of the notebook Gesture Classification_pairwise_sim_allEpoch.ipynb. Additionally, I noticed many warnings about "Maximum iterations reached" and the optimization not converging yet. Does this affect the results?
   Thank you for pointing that out, and we apologize for overlooking this error in our notebook. The notebook was originally used for data preprocessing (converting raw emotion tweets and gesture keypoints into numpy preprocessed files) and then loading the .npy files directly into the .py simulation scripts to optimize the running time of our large-scale simulation. The notebook error and warning did not affect any of our previous results. The optimization convergence warning was related to another performance measure we tested, but since we decided to use KNN for measurement, it also did not affect any of our previous results.

   Still, this was an oversight on our part and we appreciate the reviewer bringing this to our attention. To improve the readability of our code, we have modularized the code into separate scripts for data preprocessing, simulation, and results, and the notebook is no longer in use.

10. In line 523, there is a missing closing parenthesis for a corresponding opening parenthesis.
    Thank you! We have added it.

Reviewer 2

1. First, the Abstract poorly outlines the purpose of the study, the problem statement, and the study's numerical results.
   Thank you for your suggestion. We recognize the importance of outlining these key elements for our readers in the abstract. We have revised the abstract to detail the limitations of existing model

explainability methods and the empirical observations gained from analyzing our proposed learning curve methods. Given the multi-step nature of our quantitative analysis and the granular nature of our learning curve approach, we selected several prominent observations to report in our abstract.

2. While nonlinearity is indeed a characteristic of deep neural networks, the manuscript does not provide a robust theoretical framework to justify why this nonlinearity is a key insight, nor does it connect this finding to broader principles in human cognitive processing or AI development.
We thank the reviewer for highlighting our use of this term – we agree that nonlinearity is a key characteristic of DNNs, and is not in focus in this work. Our main observation in the paper is the departure from purely "straight line" learning in how DNNs come to discriminate between classes. In order to highlight this concept and not confuse the reader, we now describe our main observation as a non-monotonicity in our learning metrics (rather than "nonlinearity," which may imply reference to the formal features of an underlying learning algorithm). We also add some notes that these non-monotonic trends show up in other domains too (we reserve this discussion on p. 18 of our discussion to accommodate other suggestions to focus the introduction more).

3. The figures in the manuscript are presented as raster images of mediocre quality, which compromises their effectiveness in conveying critical information. High-quality vector images (e.g., SVG format) are essential for accurately depicting the nuances of the learning curves and the insights derived from them.
Thank you for pointing this out. For all the illustrations, we have converted them to vector format (e.g., PDF), and for all analytical plots, we have exported them at 500 PPI (which exceeds the standard 300 PPI) to ensure the quality of all the images in our manuscript. We have also uploaded all image files to the Preflight Analysis and Conversion Engine (PACE), the image quality diagnostic tool recommended by PLOS, to verify the quality of our images.

4. This research briefly touches on the application of the proposed method to multimodal tasks but does not provide a comprehensive analysis of its effectiveness in this area. It would benefit from a more detailed exploration of how the proposed learning curve analysis can be applied to multimodal tasks, with specific examples or simulations to demonstrate its effectiveness in such contexts.
Thank you for pointing this out. Yes, we originally suggested on lines 173-174 that our method could have implications for multimodal deep learning modeling, as our simulation utilizes datasets with distinguishable modalities to illustrate learning differences. For the current scope, we want to focus on how our proposed method can facilitate the understanding of distinguishable modalities (as a first step). We have updated line 173-174 accordingly. In the future, this method could contribute to understanding integrated multimodal processing, which we have added to the limitations and future research section of the conclusion.

5. The authors failed to explore the implications of temporal dynamics for different types of deep learning models, such as convolutional neural networks and transformers. The focus is primarily on recurrent neural networks, which limits the generalizability of the findings.
Thank you for your great suggestion. Yes, both CNNs and transformers can practically be used for processing time-series data, but they are less ideal than RNNs when constructing the learning curve to simulate the temporal cognitive processing of a system. We have provided more detailed

conceptual explanations in the manuscript on page 4. In essence, we focus on broader RNN architectures (including LSTMs) for constructing the learning curve, as they process signals in a sequential forward manner, which is not a characteristic of either CNNs or transformers. Additionally, we acknowledge our emphasis on recurrent architectures and, as a result, the limitation of not being able to generalize to other deep learning architectures, which addresses your next comment.

6. It would be much better, if the manuscript included a detailed comparison with other techniques, such as saliency maps, feature attribution methods, or layer-wise relevance propagation, to contextualize its contribution within the broader landscape of model interpretability. This might provide a clearer understanding of the method's strengths and weaknesses, thereby enhancing its scientific value.
   Thank you for the valuable advice. We have added Table 1 to provide a systematic overview of several major DNN explainability methods (including saliency maps, feature attribution methods, and layer-wise relevance propagation) and to compare them with the proposed learning curve approach. Based on this table, we further elaborate on the necessity of the proposed method. Overall, existing methods are either less effective for unstructured tasks, such as image and audio data, or they primarily focus on the qualitative interpretation of models. The lack of quantifiable measurements makes cross-model comparisons particularly challenging in the temporal unstructured tasks (see p. 3).

7. The manuscript does not sufficiently address the limitations of the proposed method for mapping learning curves.
   We thank the reviewer for pointing this out. We have added the limitations of both our operational illustrations and the proposed method itself in the Discussion section (p.20). Specifically, we reflected on the conceptual generalizability of our illustrations in making broader claims about the conceptual differences between language and behavioral learning. Additionally, we provided a more holistic view of the strengths and weaknesses of our learning curve method compared to other existing explainability techniques.

8. Finally, the Conclusion section is overly narrow and does not sufficiently summarize the study's numerical results and the proposed method's limitations.
   Thank you for the advice. We recognize that, given the amount of statistical analysis and significance testing results we reported in the Results section, it would be helpful to provide a holistic summary of the numerical results in the Discussion. We have added such details at the beginning of our Discussion section (p.18). Following your last comment, we have also added limitations to our Discussion section (p.20). Additionally, we further engage conceptually in the Conclusion with how cognitive science researchers have been interested in the systematic understanding of internal representations of model learning experiences and how this connects to our cognitively inspired method before linking it to model explainability research in the DNN field (p.20-21).

Reviewer 3

1. The reader is not provided with evidence of the quality of the final model. Unfortunately, the policy to stop at 20 iterations because 'the training performance shifted from underfitting to optimum to overfitting' is not technically sound. For example, the cited article by McLelland et al. used more than 2000 training iterations with an arguably simpler ML model. Absence of credible final output state undermines the empirical basis for the remaining arguments.
   Thank you for raising this concern. We agree that providing evidence for the final classification performance is important to strengthen the interpretation that the learning curves are applied to sufficiently trained models. We have added the performance report for the average of all pairwise simulations of sentence and gesture classification in our manuscript (epochs = 50) using various metrics (validation accuracy, validation loss, test accuracy, test loss, recall, precision, and F1) in S1 Table. From these results, we observe that almost all pairwise classifications (except for body & head) have been sufficiently trained, indicating that the learning curves are capturing the learned experiences, with sentence classification achieving slightly better performance than gestures. Regarding body & head, this also corresponds to the unique learning trajectory of the underlying pair, which has difficulties properly learning the signals (as revealed by our proposed method). We have discussed this observation in our manuscript (lines 604-607, p.19). The Null simulation documents the performance for randomly shuffled binary classification results.

   When we initially ran the simulation, we observed that by epoch 20, the model similarly achieved strong classification performance (over 95% in most cases) for sentence classification, and acceptable performance for gesture classification (over 65%). This is primarily because binary classification tasks are simpler than the multiclass tasks we commonly encounter. Additionally, our demonstration mainly focuses on the differences in learning experiences across tasks, which tend to occur in the early stages of training (early epochs).

   We acknowledge that gesture learning is more difficult, and 20 epochs may not be sufficient. Therefore, we have extended our simulation in the main text to 50 epochs. However, we have retained the visualization of 20 epochs in S3 Fig. to show that the patterns remain largely consistent. Additionally, we have included model performance and visualizations for 100 epochs with 10 runs of pairwise gesture classification simulations in S1 Table. and S4 Fig. to further support our claim that most differences in learning experiences emerge during the early stages of training, and that there are no substantive qualitative differences between epochs 50 and 100.

2. As for statistical analysis, I would suggest to show significance with respect to a reasonable null hypothesis such as, for example, a random superposition of emotion or gesture states, instead of simply showing that all trials have a similar trend on a short training cycle.
   This is a great suggestion! In addition to our previous hypothesis testing on how the learning curves differ for the two datasets, we also conducted 20 additional simulation runs using the superposition of emotion and gesture states (i.e., reshuffled dataset labels) as a more meaningful null hypothesis. The results are reported alongside other significant tests in Tables 2 (p.14) and Figures 6-9. We also included further discussion on how the simulated results differ from the learning curve of superposition states (p.13-17), which highlights the additional value of understanding the underlying signal processing of our learning curve method.

3. Conceptually, the computation of the metrics is quite intricate -- in what way do they add clarity to an opaque RNN model? A constructive analysis, perhaps by way of a simple example, could help understand this aspect better.

   Thank you for raising this valuable concern. While we acknowledge that our analysis involves multiple steps—from learning curve construction to quantifying the curves to hypothesis testing—the main component of our method, learning curve construction, is relatively straightforward and intuitive (i.e., plotting the performance trajectories across timesteps and epochs). We added a flowchart to help readers better understand our procedures (Fig 2, p.7).

   The four instances we introduced here to compare our constructed curves are simple descriptive statistics we define to simplify the comparison of these multidimensional artifacts (see p.11). However, researchers have the flexibility to decide how they want to analyze the learning curve. They can use all four descriptive statistics we propose, or choose to use only one or two of our proposed instances, or define other measures (such as fitting a polynomial regression or simply qualitatively interpreting the curve plots) that suit their use case.

   We have clarified these potential uses of our proposed method (p.11, lines 352-356) and hope this can alleviate the reviewer's concern regarding the complexity of computing our proposed metrics.

4. Overall, the methodology could be both more concise and more detailed. I would recommend using a more structured approach and adhere to standard practices, e.g. architecture diagrams for neural networks.

   Thank you for the great suggestion. We made several efforts to streamline both our methods and results sections:

   1) We have added two figures to better clarify our methodology. These include a flowchart from model training to learning curve construction to the quantification of the four measures in Figure 2, and a diagram of the RNN-LSTM architecture in Figure 4.
   2) We also streamlined our last section "Quantifying and Analyzing the Learning Curves" in the Methods section (see p.11-13) by moving some of the definitions of the quantitative measures to the introduction, and keeping the rest more concise.
   3) Additionally, we restructured our Results section. It now presents a meta-results table (Table 2) organizing all the significant tests we have conducted, with each metric subsection referring back to the values in the table.

   We hope these changes have made our methodology more structured and digestible.

5. The presentation uses a narrative style, requiring a considerable effort to follow and identify key original contributions. For example, a clear and concise statement about all four metrics being the main methodological contribution appears only on page 15.

   Thank you for pointing this out. We recognize the importance of presenting an easy-to-follow structure in our manuscript for our audience. Following your advice, we first provide a concise definition of our four metrics in the Introduction (see p.5-6, lines 141-156) and tidy up the conceptualization and definition of these metrics in the "Quantifying and Analyzing the Learning Curves" section (see p.11-13)

We also acknowledge that the narrative style made it harder for the audience to follow our argument. Similar to our previous response, we restructured the Results section by presenting a meta-results table that includes all significant tests, with each metric subsection referring back to the corresponding values in the table.

We hope these changes help improve the reading experience for our target audience.

6. The research is not directly related to neuroscience or psychology research and as a result, large portions of introductory discussion is irrelevant for this work.
   Thank you for raising this concern. We acknowledge that this research does not directly compare the model's learning experience to behavioral or neuroscience responses in humans. Nonetheless, we believe our proposed method is inspired by a similar paradigm in cognitive science, where researchers have been exploring model representations to better understand human psychology over the past decade. We also appreciate your suggestion that an overly lengthy discussion of neuroscience or psychology research could distract our target audience. Following your advice, we have streamlined the Introduction while ensuring that the Discussion and Conclusion sections still engage with the relevant neuroscience and psychology implications of our proposed methods.

7. Lastly, the authors are rightly concerned by over-reliance on AI for decision making, but wrongly attribute the fault to complicated models. The most common causes of ethical issues reside in flawed datasets, not complicated models (assuming sound technical execution and fair play).
   Thank you for pointing this out. We agree that, given the nature of our paper, which focuses on proposing methods to understand multidimensional embeddings, we have clarified our concern to be more explicitly related to the challenge of interpreting the multidimensional representations of DNNs, rather than broadly discussing the black box issue of DNNs. We have also highlighted that the concern over over-reliance on AI for decision-making arises more from issues such as "relying on training data that does not generalize well for the target tasks" (see page 2, lines 11-14).

Reviewer 4

1. Abstract: Describes what "natural language processing" means and stands for (NLP). Why didn't you do the same with Deep Neural Networks (DNN)?
   We have changed "NLP" to "sentence classification" and provided the full name for DNN to improve clarity.

2. The current study:
   To conduct such a study and unpack "DNN learning curves, ... It was not clear what some understandings are in this item:
      i) Ability to process time series, ...
      ii) Capture temporal dependencies through their recurrent connections, ...
      iii) Use as a basis changes in "predictability" or "information", ...
   This information was not clear.
   Subjectivity in the format of how this process occurs.

My suggestion is a more detailed description of the generation of the series and how this information is captured.
We thank the reviewer for noting this issue. Instead of using conceptual terms like “predictability”, we have opted for the more direct definition, “classification performance between embeddings and outputs” (p.5, lines 123-124), for clarity. Additionally, we have provided a more detailed description of how RNNs generate a series of embeddings and what signals are captured at each time step to help our readers better understand the construction and conceptualization of our proposed method (see p.5, lines 126-128).

3. Feedforward network:
A normalization was applied to the input. It was conducted in every time interval. Normalization was applied to the input to convert information, …
There are many details in the text about normalization, however, the technique is vague. The text does not describe that normalization is used to transform input data so that it is within a domain (specific range) with a focus on improving the network's performance. We have normalization for training, to prevent or fade gradients to improve performance and we also have types of normalization.
The question goes in this direction. What is the objective (to describe)? What is the type (to describe)? What is the domain of normalization? 0 and 1?
I understand that this can be done. It would help to better understand this topic.
Thank you for pointing it out. We have provided more details regarding our batch normalization techniques on page 10, lines 278-284. In essence, the objective here is to enhance training stability and speed up convergence by normalizing across timesteps within each batch (i.e., each timestep is treated independently, and normalization is applied across all data points in the batch for each timestep) and standardizing the inputs to have zero mean and unit variance within each batch of data using the BatchNorm1d layer.

4. Quantifying performance across epochs
The description of this topic was unclear on some points:
   i) We tested several regression techniques. Which ones? Technically, we have several. It was a simple regression, logistic, polynomial, Ridge, Poisson, Support Vectors, Bayesian, ... I believe you can tell us which regression was used.
   ii) The regression coefficients. Which ones?
Thank you for pointing these out. We were using a more narrative-based style to document the measures we initially attempted before deciding to use the four simpler quantitative metrics that we ultimately employed. We found this descriptive statistics approach (i.e., the four introduced metrics) to be a much more intuitive way to quantify the multidimensional curves than using regression coefficients. We acknowledge that the previous wording may have been somewhat distracting or confusing for the audience, as they were not fully explained in detail. In response, we have removed the part related to the regression techniques (which we didn’t end up using in the manuscript) from the “Quantifying and Analyzing the Learning Curves” section in our Methods (p.11-13), emphasizing a clearer conceptualization and definition of the four metrics used in our analysis.

   iii) Simple summary statistics. Do you mean average? What is this simple summary? I understand that you can add details.

Thank you for your comment. The term “simple summary statistics” refers to the four metrics (*start*, *max*, t_{max}, and *end – start*) we proposed in this study. We recognize that using “summary statistics” and “descriptive statistics” interchangeably could be confusing, and have therefore replaced all instances with “descriptive statistics” for consistency.

iv) What would explain the transition of environment (Python to R)? What is Python limited in?
Thank you for pointing this out. Initially, we attempted to conduct both the simulation and result analytics in Python, but ultimately switched to R for the analytics portion due to its seamless integration and better usability for statistical analysis. For our analysis, R offers cleaner result reporting and more powerful plotting capabilities, especially for our multidimensional graphs. We have also explained this consideration on p.13, lines 407-409.

5. Results
Again, I repeat here. We tested a linear model. Which one?
Thank you for pointing this out. We used multiple regression models for our significance tests. We have organized all the models we ran into a meta-results table (see Table 2) and added a paragraph clearly explaining the models we used and what they conceptually examine (see p.13, lines 420-430).

6. Discussion
Check for non-linearity (it's in the text).
I understand that you can add details on how you checked for non-linearity.
We thank the reviewer for highlighting this. We agree that nonlinearity is a key characteristic of DNNs, but it is not the focus of this work, and there are possible statistical approaches to check for nonlinearity. We want to emphasize that our main observation in the paper is the departure from purely “straight-line” learning in how DNNs come to discriminate between classes. To avoid confusing the reader, we now describe our main observation as” non-monotonicity” in our learning metrics (rather than “nonlinearity”, which may imply a reference to the formal features of the underlying learning algorithm). Further details on non-monotonicity can be found in the Discussion section on p. 18.

7. Domain distinctions
To characterize the smoothness of a curve from the point of view of autocorrelation, it is very common to use a model that takes the scale self-affinity, a property of looking at information from the point of view of time scales, for example the Detrended Fluctuation Analysis method, Peng 1994.

For the information described “Smoother self-correlated signals”, I actually didn’t understand. Another point is also related to “self-correlated spatial information contained in the gesture performance. I believe more details can be added to this description.
We thank the reviewer for sharing the resource for statistically assessing curve smoothness, which is an excellent toolkit to help researchers make claims about smoothness as a statistical property of results. We would like to clarify that, in our case, smoothness is a theoretical concept used to describe the learning experience of gestures. This learning experience is autocorrelated due to the fact that body movements result from the coordinated contraction and relaxation of muscles, where signals produced at later timesteps are derived from previous timesteps with relatively high autocorrelation (p.19, lines 621-622). We recognize that the use of the term “smoothness” could cause confusion with its statistical

interpretation and have removed it from the manuscript. Additionally, we provide further clarification on the “spatial information contained in the gesture performance” in the footnote of p.19. Specifically, this refers to “the keypoint coordinates in the gesture dataset, which can vaguely help the model distinguish between gesture classes.”
