# Supplementary material for: Mapping the learning curves of deep learning networks
Source: PLoS Comput Biol. 2025 Feb 10;21(2):e1012286. doi: 10.1371/journal.pcbi.1012286 (PMC11841907; doi:10.1371/journal.pcbi.1012286)
Supplement: Attachment — Submitted filename: PLOS Computational Biology_FINAL_OPINION.pdf [file pcbi.1012286.s008.pdf]

PLOS Computational Biology

About the article: **PCOMPBIOL-D-24-01100** "Mapping the Learning Curves of Deep Learning Networks"

I have verified that all the suggestions sent were valued and accepted by the authors. In a coherent manner, all the questions were answered and added to the main article.

I understand that the article has gained more clarity and that now potential readers will be able to understand the proposal of the work more properly.

I would like to take this opportunity to state here that I am in favor of publishing the final version of the article entitled "Mapping the Learning Curves of Deep Learning Networks".
